# Predictive value of EGFR overexpression and gene amplification on icotinib efficacy in patients with advanced esophageal squamous cell carcinoma

**DOI:** 10.18632/oncotarget.8271

**Published:** 2016-03-22

**Authors:** Xi Wang, Haitao Niu, Qingxia Fan, Ping Lu, Changwu Ma, Wei Liu, Ying Liu, Weiwei Li, Shaoxuan Hu, Yun Ling, Lei Guo, Jianming Ying, Jing Huang

**Affiliations:** ^1^ Department of Medical Oncology, Cancer Hospital, Chinese Academy of Medical Sciences & Peking Union Medical College (CAMS & PUMC), Beijing, China; ^2^ Department of VIP Medical Oncology, Cancer Hospital, Chinese Academy of Medical Sciences & Peking Union Medical College (CAMS & PUMC), Beijing, China; ^3^ Department of Medical Oncology, The First Affiliated Hospital of Zhengzhou University, Zhengzhou, Henan, China; ^4^ Department of Medical Oncology, The First Affiliated Hospital of Xinxiang Medical College, Xinxiang, Henan, China; ^5^ Department of Medical Oncology, Chifeng City People's Hospital, Chengfeng, Inner Mongolia, China; ^6^ Department of Medical Oncology, The Fourth Hospital of Hebei Medical University, Shijiazhuang, Hebei, China; ^7^ Department of Medical Oncology, Cancer Hospital of Henan Province, Zhengzhou, Henan, China; ^8^ Department of Pathology, Cancer Hospital, Chinese Academy of Medical Sciences & Peking Union Medical College (CAMS & PUMC), Beijing, China

**Keywords:** EGFR, overexpression, amplification, icotinib, esophageal cancer

## Abstract

This study aimed to search for a molecular marker for targeted epithelial growth factor receptor (EGFR) inhibitor Icotinib by analyzing protein expression and amplification of *EGFR* proto-oncogene in esophageal squamous cell carcinoma (ESCC) patients.

Immunohistochemistry and fluorescence *in situ* hybridization (FISH) was used to assess EGFR expression and gene amplification status in 193 patients with ESCC. We also examined the association between EGFR overexpression and the efficacy of a novel EGFR TKI, icotinib, in 62 ESCC patients.

Of the 193 patients, 95 (49.2%) patients showed EGFR overexpression (3+), and 47(24.4%) patients harbored EGFR FISH positivity. EGFR overexpression was significantly correlated with clinical stage and lymph node metastasis (p<0.05). In addition, EGFR overexpression was significantly correlated with EGFR FISH positivity (p<0.001). Among the 62 patients who received icotinib, the response rate was 17.6% for patients with high EGFR-expressing tumors, which was markedly higher than the rate (0%) for patients with low to moderate EGFR-expressing tumors (*p*=0.341). Furthermore, all cases responded to icotinib showed EGFR overexpression.

In conclusion, our study suggests that EGFR overexpression might potentially be used in predicting the efficacy in patients treated with Icotinib. These data have implications for both clinical trial design and therapeutic strategies.

## INTRODUCTION

Esophageal cancer is the sixth most common cause of cancer death all over the world [1]. Despite major advances in surgical procedures and chemoradiotherapy, it remains one of the most aggressive and fatal malignancies with an overall 5-year survival rate less than 20% [2]. Most patients in China present with an advanced or metastatic stage and the dominating type is esophageal squamous cell carcinoma (ESCC). Cytotoxic chemotherapy (cisplatin combined with fluorouracil or paclitaxel) remains the main stay of treatments for patients with metastatic ESCC [3–5], despite the fact that targeted therapy has already played an important role in other cancer types such as non-small-cell lung cancer, breast cancer, colon cancer and gastric cancer etc. However, molecular targeted therapy would likely become a promising therapeutic approach for the treatment of ESCC [6].

The epidermal growth factor receptor (EGFR) gene encodes a membrane glycoprotein, which is responsible for EGFR signaling up-regulation. EGFR gene is involved in a wide variety of malignancies such as non-small cell lung cancer, colon cancer and head-and-neck squamous cell carcinoma [7]. EGFR expression was usually associated with more advanced tumor stage as well as reduced overall survival for patients with esophageal and esophagogastric junction adenocarcinomas [8]. It was found that approximately 50-70% of esophageal tumors express EGFR protein when examined using immunohistochemistry (IHC), while 15-28% of specimens also exhibit *EGFR* gene amplification when examined using fluorescence *in situ* hybridization (FISH) [9]. However, the relationship between the EGFR overexpression or gene amplification and the clinicopathologic features remains unclear in ESCC.

To address the association of EGFR overexpression and gene amplification with clinical characteristics of ESCC patients, we evaluated 193 ESCC samples in this retrospectrive study. Icotinib is an oral, selective EGFR TKI which showed non-inferior efficacy to gefitinib in the treatment of non-small cell lung cancer (NSCLC) [10]. It has been approved by the State Food and Drug Administration in China for treatment of NSCLC patients. We also assessed EGFR overexpression and gene amplification in 62 patients who received icotinib to identify ESCC subgroups who might benefit from EGFR inhibitor therapy.

## RESULTS

### Clinical characteristics

The clinicopathological characteristics of 193 ESCC patients were summarized in Table 1. According to the result, the majorities were males (85.5%), with a median age of 60 years (range: 43 to 78). 56.5% (109/193) of the tissues had been obtained by biopsy and 43.5% (84/193) of the tissues by surgical resection. With regards to the proportion in tumor differentiation, grade 3 was observed in approximately half of the total subjects (48.2%), followed by grade 2 (41.5%) and grade 1 (10.4%). 83.4% (161 out of 193 patients) had lymph node metastases.

**Table 1:** Summary of demographic information.

Clinical characteristics	Case	EGFR expression			EGFR FISH^a^		
		0~2+	3+	P value	Negative	Positive	P value
Age (year)							
<=65	149	76	73	0.907	109	40	0.138
>65	44	22	22		37	7	
Gender							
Male	165	88	77	0.085	126	39	0.574
Female	28	10	18		20	8	
Histologic grade							
G1	20	10	10	0.421	14	6	0.737^b^
G2	80	45	35		60	20	
G3	93	43	50		72	21	
Clinical stage							
I	7	6	1	0.045^b^	6	1	0.146^b^
II	30	16	14		24	6	
III	81	46	35		66	15	
IV	75	30	45		50	25	
Lymph node metastasis							
No	32	22	10	0.026	27	5	0.208
Yes	161	76	85		119	42	

a Fluorescence In Situ Hybridization

b Tested by Fisher's exact test

### EGFR protein expression

**Figure 1:**
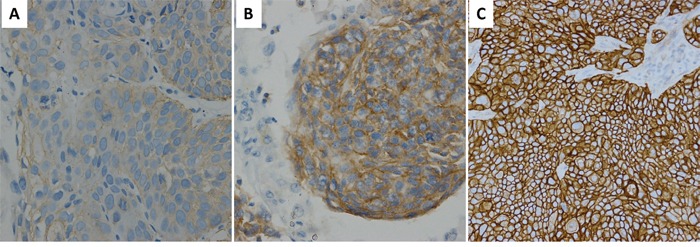
EGFR IHC staining in ESCC EGFR IHC staining of 1+ **A.** 2+ **B.** 3+ **C.** original magnification 200X.

Among 193 cases, EGFR was expressed in 191 cases (99.0%). Ninety-five (49.2%) cases were scored 3+ for EGFR staining and interpreted as EGFR overexpression, 83 (43.0%) cases scored 2+ and 13 tumors (6.7%) with low level (1+) immunoreactivity were interpreted as no EGFR overexpression. No EGFR immunoreactivity was detected in 2 (1.0%) of the tumors. In ESCC, EGFR overexpression was significantly correlated with clinical stage and lymph node metastasis (p<0.05), but not with other independent variables (Table 1). Figure 1 illustrates EGFR Immunohistochemistry staining in ESCC.

### EGFR FISH analysis

**Figure 2:**
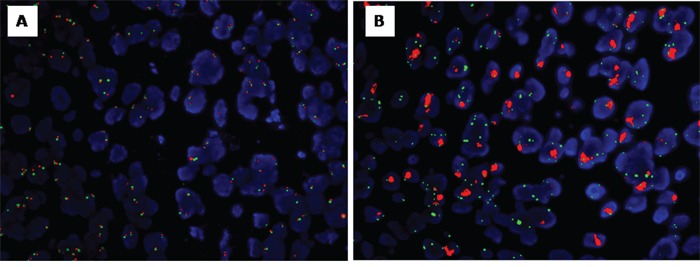
EGFR FISH analysis EGFR FISH: negative **A.** the EGFR FISH: positive **B.**

According to the EGFR FISH analysis, gene amplification was present in 26 patients (13.5%), high polysomy in 21 (10.9%) and other conditions in 146 (75.6%). Therefore, 47 patients (24.4%) were categorized in the EGFR FISH-positive group and 146 patients (75.6%) in the EGFR FISH-negative group. The prevalence of EGFR FISH positivity in this patient population was not correlated with any clinical parameter (Table 1). Figure 2 illustrates EGFR FISH analysis.

### Correlation between EGFR overexpression and EGFR FISH positivity

FISH analysis revealed that EGFR FISH positivity was detected in 34.7% (33/95) of the patients with EGFR protein overexpression. For the patients with lymph node metastasis, 52.8% (85/161) cases showed EGFR protein overexpression and 26.1% (42/161) demonstrated EGFR FISH positivity. For the patients with distant metastasis, 60.0% (45/75) showed EGFR protein overexpression and 33.3% (25/75) demonstrated EGFR FISH positivity (Table 1). Table 2 provides information regarding EGFR overexpression and EGFR FISH positivity among the target population. EGFR FISH positivity was associated with EGFR protein overexpression (p< 0.01). For 15 cases with EGFR protein expression negative or 1+, EGFR FISH results were all negative.

**Table 2:** Correlation between EGFR expression and EGFR FISH^a^ results

EGFR expression	EGFR FISH			P value
	case	Negative	Positive	
0	2	2	0	0.003^b^
1+	13	13	0	
2+	83	69	14	
3+	95	62	33	

a Fluorescence In Situ Hybridization

b Tested by Fisher's exact test

### Tumor response to icotinib

The clinicopathological features of 62 ESCC patients who received icotinib were summarized in Table 3. The majority of patients were males (80.6%). The median age of patients was 60 years. There was one patient achieving a complete response and eight patients achieving partial responses to icotinib. Twenty patients had stable disease and 33 patients developed progressive disease on icotinib therapy. Among the 62 patients evaluated for EGFR expression, 11 patients (17.7%) had low or moderate (1+ or 2+) EGFR expression and fifty-one (82.3%) were highly positive for EGFR. The response rate was 17.6% for patients with high EGFR-expressing tumors, which was markedly higher than the rate (0%) for patients with low to moderate EGFR-expressing tumors (*p*=0.341), although this was not statistically significant (Table 4).

**Table 3:** Correlation of EGFR expression and FISH^a^ and the clinical features

Characteristics	Patients (n=62)	EGFR overexpression (n=51)		EGFR FISH (n=22)	
	No. (%)	No./Subgroup (%)	P value	No./Subgroup (%)	P value
Age (year)					
<=65	47 (75.8)	36/47 (76.6)	0.052^b^	17/47 (36.2)	0.842
>65	15 (24.2)	15/15 (100.0)		5/15 (33.3)	
Gender					
Male	50 (80.6)	39/50 (78.0)	0.102^b^	18/50 (36.0)	1.000^b^
Female	12 (19.4)	12/12 (100.0)		4/12 (33.3)	
Performance status					
0-1	59 (95.2)	48/59 (81.4)	1.000^b^	22/59 (37.3)	0.546^b^
2	3 (4.8)	3/3 (100.0)		0/3 (0.0)	
Smoking					
Never	23 (37.1)	22/23 (95.7)	0.042^b^	8/23 (34.8)	0.929
Ever	39 (62.9)	29/39 (74.4)		14/39 (35.9)	
Histologic grade					
G1	6 (9.7)	5/6 (83.3)	0.305^b^	3/6 (50.0)	0.004^b^
G2	17 (27.4)	12/17 (70.6)		11/17 (64.7)	
G3	39 (62.9)	34/39 (87.2)		8/39 (20.5)	
No. of previous chemotherapy					
None or 1	43 (69.4)	41/43 (95.3)	<0.001^b^	15/43 (34.9)	0.882
2 or more	19 (30.6)	10/19 (52.6)		7/19 (36.8)	
Disease extent					
Locally advanced	6 (9.7)	3/6 (50.0)	0.063^b^	2/6 (33.3)	1.000^b^
Metastatic	56 (90.3)	48/56(85.7)		20/56 (35.7)	

a Fluorescence In Situ Hybridization

b Tested byFisher's exact test

**Table 4:** Responses of icotinib therapy in patients according to total EGFR expression and EGFR FISH^a^

Tumor status	No. of evaluated Patients	Complete responses and partial responses		P value	Stable disease	
		No.	%		No.	%
Total	62	9	14.5		20	32.3
EGFR expression						
1+, or 2+	11	0	0	0.341^b^	3	27.3
3+	51	9	17.6		17	33.3
EGFR FISH^c^						
Negative	39	4	10.3	0.713^b^	14	35.9
Positive	22	4	18.2		6	27.3
Unknown	1	1	100		0	0

a Fluorescence In Situ Hybridization

b Tested by Fisher's exact test

c one case was exclude because the result of EGFR FISH couldn't be detected

Among the 9 responsive cases, 7 cases had ≥50% tumor cells showing strong cytoplasmic or/and membranous reactivity, one case with 40% and another case with 10%. Also, among the 9 response cases, 7 were poorly differentiated, one was moderately differentiated and one was well differentiated. The two moderately or well differentiated samples were from surgical resection of primary tumor.

Among the 61 patients evaluated for EGFR FISH, 39 cases (63.9%) were FISH negative, and 22 cases (36.1%) were FISH positive, including 9 cases with high polysomy and 13 cases with gene amplification. Among the 22 cases with EGFR FISH positivity, 4 cases achieved partial response. Two of the four cases with partial response were identified as high polysomy of EGFR gene, whereas the other two were amplification of EGFR gene. The response rate of FISH-positive patients was 18.2%, higher than that of FISH-negative patients with a response rate of 10.3%, although this was not statistically significant (Table 4). For the remaining patient with CR, the FISH result was unknown.

## DISCUSSION

Despite the current trend towards personalized medicine, there are no targeted biologic agents which are applicable to ESCC. The prevalence of EGFR expression in ESCC patients has rendered it an appealing candidate for targeted therapy, yet whether the overexpression or gene amplification of EGFR could predict the response to EGFR TKI therapy in ESCC patients remains unclear. To the best of our knowledge, the present study is the largest case series focusing on the analysis of predictive biomarkers for EGFR TKI therapy in advanced ESCC. Our study reported the response rate was 17.6% for ESCC patients with high EGFR-expressing tumors, which was markedly higher than the rate (0%) for patients with low to moderate EGFR-expressing tumors. Furthermore, all the cases responding to icotinib showed EGFR overexpression, suggesting a trend towards improved efficacy in the subgroup of patients with EGFR overexpression or amplification.

Previous studies have reported modest treatment benefit of EGFR TKI in unselected esophageal cancer patients. One phase II clinical trial showed that EGFR-TKI erlotinib had activity in a small number of patients with ESCC, with partial responses in 15% patients (2 of 13) and stable disease in 54% (7 of 13) patients with squamous carcinoma [11]. The placebo-controlled phase III COG trial only demonstrated a marginal PFS benefit of gefitinib over placebo as second-line therapy for unselected esophageal cancer patients (median PFS, 1.57months vs. 1.17 months, p=0.020) [12]. Compared with these findings, more encouraging treatment outcome was observed in our cohort of ESCC patients with predominant EGFR overexpression or amplification, suggesting that EGFR-targeted therapy in ESCC is promising and worthy of further exploration. Moreover, biomarker analysis was incorporated into our study, revealing the potential role of EGFR overexpression/amplification in predicting treatment efficacy.

In this study, EGFR protein overexpression was found in 49.5% of the ESCC cases, and 34.7% of the overexpressing tumors showed EGFR FISH positivity. EGFR protein overexpression was significantly associated with EGFR FISH-positivity. These findings were in line with previous reports [9, 13]. While EGFR overexpression was associated with clinical stage and lymph node status, there was no association between EGFR/FISH-positivity and clinical stage. Compared with previous studies [9, 13], our study retrospectively analyzed the association between EGFR overexpression and gene amplification in a larger number of ESCC patients, including metastatic cases being firstly analyzed.

The predictive value of EGFR over-expression in esophageal cancer receiving EGFR-TKI therapy was previously reported by Janmaat ML, et al [13]. In their study, 9 patients with high EGFR expression (IHC 3+) demonstrated a significantly better disease control rate than the remaining 15 patients (66.7% vs. 6.7%, P=0.002). However, it is noteworthy that their study included only 6 ESCC patients, thus the predictive value of EGFR expression in ESCC patients were not fully elucidated. Our study confirmed that EGFR overexpression was also a potential predictive factor for the response to EGFR-TKI in ESCC because all the response cases in our trial were identified as IHC 3+. Among the 9 response cases in our study, the two moderately and well differentiated cases were both from primary surgical tissues, while no re-biopsy was conducted after recurrence. The other 7 cases were all histological poor differentiation. Therefore, poor differentiation may also be a predictor for clinical outcome to icotinib in ESCC patients in the future.

Recently, researchers are working on a wide variety of strategies to achieve personalized cancer care in treating ESCC. Anti-EGFR therapies might be considered as a potential alternative in treating ESCC. Our results suggest that combined analyses with both IHC and FISH could help identify a group of patients who would benefit from anti-EGFR therapies. More promise in targeting the EGFR pathway in ESCC may come from monoclonal antibodies that can block the binding of ligands to the receptor. Encouraging response rates and median survival have been reported from a randomized phase 2 trial of cetuximab plus cisplatin-5-FU versus cisplatin-5-FU alone in first-line metastatic ESCC patients [14].

Although EGFR overexpression or amplification was demonstrated to be an effective predictor of treatment outcome, the response rate in the selected patients was still unsatisfactory. Thus, future research should focus on the exploration of additional biomarkers to optimize the selection of patients responsive to EGFR-TKI. One of the important mechanisms underlying resistance to EGFR-targeted therapy is the aberrant activation of its two main downstream signaling pathways: RAS/RAF/MAPK pathway and PI3K/AKT/mTOR pathway. Janmaat ML et al. reported that K-ras mutation was identified in two (8.7%) of 23 esophageal cancer patients treated with gefitinib and was associated with early disease progression [13]. In another retrospective study of 32 ESCC patients treated with nimotuzumab (an anti-EGFR monoclonal antibody) combined with radiotherapy or chemoradiotherapy, the combination of EGFR and Akt protein levels was found to be a predictor of treatment outcome [15]. Interestingly, significantly improved survival was observed in the subgroup of patients with high EGFR/low Akt expression but not in the EGFR high/Akt high patients, suggesting that the aberrant activation of Akt (as evidenced by its high expression) by other signals may result in resistance to EGFR-targeted therapy. Furthermore, the aberrant activation of several bypass pathways, including HER-2, HER-3 and C-MET pathways, also plays an important role in inducing resistance to EGFR TKI therapy [16]. Further knowledge of these molecular mechanisms may assist in the development of new therapeutic strategies to enhance treatment efficacy of EGFR-targeted therapy.

In conclusion, we evaluated the prevalence of EGFR protein expression, EGFR/FISH-positivity status, and the efficacy of icotinib in ESCC. Given the potential efficacy observed and its correlation with the molecular marker characteristics, our findings would provide some insights in the use of icotinib in ESCC, patients in the future.

## MATERIALS AND METHODS

### Patients and tumor samples

This study was performed retrospectively. Tumor specimens were collected from 6 Chinese clinical centers, reviewed and evaluated from 193 patients with histologically confirmed esophageal squamous cell carcinoma by two pathologists. The World Health Organization Classification of Tumors was used for histologically grading. The tumors were staged according to the tumor-node-metastasis (TNM) classification of the American Joint Committee on Cancer (AJCC 7^th^). This retrospective study was approved by the institutional ethics committee of Cancer Hospital, Chinese Academy of Medical Sciences, and the waiver of informed consent was obtained.

### Immunohistochemistry (IHC)

IHC detection of EGFR was carried out in all 193 patients with ESCC, using the pre-diluted CONFIRM anti-EGFR (5B7) Rabbit monoclonal primary antibody (Ventana Medical Systems/Roche Diagnostics, Tucson, AZ, USA) with a Ventana Ultraview Universal DAB detection kit in Ventana Benchmark XT stainer (Ventana/Roche). EGFR IHC was scored independently by two pathologists blinded of the clinical information using the scoring scheme proposed as follows [13]: 0, no staining; 1+, faint cytoplasmic or/and membranous reactivity; 2+, moderate cytoplasmic or/and membranous reactivity; 3+, strong cytoplasmic or/and membranous reactivity in ≥10% of tumor cells. Tumors with a score 3+ were interpreted as high expression (EGFR overexpression), while tumors with a score 1+ or 2+ were interpreted as no EGFR overexpression. The percentage of tumor cells exhibiting strong cytoplasmic or/and membranous reactivity was marked for all 3+ cases.

### Fluorescence *in situ* hybridization

The *EGFR* gene copy number and Chr-7 were detected by Fluorescence *in situ* Hybridization (FISH) assay with Vysis EGFR/CEP 7 FISH Probe Kit (Abbott Molecular), according to manufacturer's instruction. *EGFR* gene copy number, Chr-7 number and *EGFR*/Chr-7 copy number ratio were assessed.

According to the criteria which were reported previously, signals from at least 100 cancer cell nuclei were counted and the EGFR gene copy number was classified into six subgroups: ① disomy (2 or less copies in more than 90% of cells); ② low trisomy (2 or less copies in 40% or more of cells, 3 copies in 10%–40% of cells, 4 or more copies in less than 10% of cells); ③ high trisomy (2 or less copies in 40% or more of cells, 3 copies in 40% or more of cells, 4 or more copies in less than 10% of cells); ④ low polysomy (4 or more copies in 10%–40% of cells); ⑤ high polysomy (4 or more copies in 40% or more of cells); ⑥ gene amplification (defined by the presence of tight EGFR gene clusters and a ratio of EGFR genes to chromosome of 2 or more, or 15 or more copies of EGFR per cell in 10% or more of the cells analyzed). EGFR FISH positivity was considered as high polysomy or gene amplification. FISH analysis was undertaken for all cases of 193 patients with ESCC.

### Icotinib therapy

We retrospectively reviewed 62 consecutive ESCC patients who received icotinib (250mg/time, 3 times daily) between December 2013 and May 2015 for at least 4 weeks by oral administration and were evaluated for responses. Of the 62 patients, 4 patients had no prior exposure of systemic chemotherapy, 39 patients have received one prior chemotherapy regimen, and the other 19 patients have received at least two previous chemotherapy regimens. Follow-up information was provided either by the referring clinicians, or obtained directly from patients and their family members. The date of last follow up was May 16, 2015. Objective response rate (ORR), disease control rate (DCR) were all evaluated according to Response Evaluation Criteria in Solid Tumors (RECIST Version 1.1).

### Statistical analysis

Statistical analyses were performed using SPSS 22.0 (SPSS Inc., USA). Pearson's χ2 test or Fisher exact test was used for analysis of the relationship between clinicopathological features and EGFR-IHC/FISH status, and the correlation between IHC and FISH results. All analyses were two-tailed, and a P-value of less than 0.05 was regarded as statistically significant.
